# A bibliometric study related to the treatment of myocardial ischemia-reperfusion Injury

**DOI:** 10.1186/s13019-024-02924-3

**Published:** 2024-07-01

**Authors:** Jie Feng, Leilei Han, Yunman Liu, Kai Li, Yanqing Wu

**Affiliations:** 1 Department of Cardiology, The Second Affiliated Hospital, Jiangxi Medical college, Nanchang University, Nanchang, 330006 China; 2 Department of Cardiovascular Medicine, The Second Affiliated Hospital, Jiangxi Medical college, Nanchang University, No. 1 Minde Road, Nanchang, Jiangxi 330006 China

**Keywords:** Bibliometrics, MIRI, Treatment, Visual analysis, Heart regeneration

## Abstract

**Background:**

Myocardial ischemia-reperfusion injury (MIRI) is defined as the restoration of blood flow to the myocardium after a brief interruption of blood supply, causing more severe damage to the ischemic myocardium. However, currently, reperfusion therapy is the preferred therapy for ischemic cardiomyopathy, which undoubtedly causes MIRI, and thus it has become a challenging issue affecting the prognosis of coronary artery disease.

**Methods:**

A search was conducted in the Web of Science Core Collection database for papers relevant to MIRI therapy published between 1 January 2000 and 1 October 2023. Bibliometric analyses were performed using VOSviewer and CiteSpace to elucidate the progress and hotspots.

**Results:**

3304 papers from 64 countries, 2134 research institutions and 13,228 authors were enrolled in the study. Of these, China contributed the most papers and had the biggest impact, while the United States had the most extensive partnership. The Fourth Military Medical University was the primary research institution. The most valuable authors include Chattipakorn, Nipon, Chattipakorn, Siriporn c, Yang, Jian and Yang, Yang.

**Conclusion:**

Over the past 20 years, research on MIRI therapies has made significant strides. Further studies are necessary to explore the interactions between various therapeutic options. Future investigations will emphasize nanocarriers, cardiac regeneration, and stem cell therapies. Our study identifies MIRI research hotspots from a bibliometric perspective, forecasts future trends, and offers fresh insights into MIRI therapy research.

## Introduction

Globally, ischemic heart disease stands as a significant contributor to mortality in patients. Recent figures from the World Health Organization illustrate that in 2019 alone, ischemic heart conditions were responsible for approximately 8.86 million deaths, constituting 16% of total global fatalities [1]. The predominant manifestation of this disease is acute myocardial infarction, marked by the death and apoptosis of myocardial cells, leading to substantial heart tissue damage. Notably, myocardial cells are categorized as permanent cells, demonstrating limited capacity for proliferation, regeneration, and repair. Presently, the frontline treatment for acute myocardial infarction involves reperfusion therapies such as thrombolysis or percutaneous coronary intervention (PCI). These treatments are designed to swiftly restore blood flow to the heart, minimize the area affected by the infarction, and enhance the overall clinical outlook [2]. Nevertheless, the interim recovery of blood flow to the ischemic myocardium can paradoxically expand the zone of cardiac injury [3]. This adverse effect, known as myocardial ischemia-reperfusion injury, potentially diminishes the therapeutic benefits in managing coronary heart disease, and is also referred to as lethal reperfusion injury [4]. The concept of myocardial reperfusion injury was initially introduced by Jennings et al. in the early 1960s [5].

Over the past two decades, there has been notable advancement in understanding myocardial ischemia-reperfusion injury (MIRI) and its related treatment methodologies. Despite reperfusion therapy being substantially advantageous for certain areas of the myocardium, the ischemia-reperfusion process can trigger cell death and enlarge the infarct size. Recent explorations reveal that post-reperfusion therapy mortality rates hover around 10%, with the prevalence of heart failure nearing 25% [6–8]. Correspondingly, animal model studies on acute myocardial infarction indicate that up to 50% of the damage can be attributed to reperfusion injury. Several strategies to mitigate lethal reperfusion injury have been validated; however, their integration into clinical practice remains challenging [9].

With the dramatic surge in medical research output over the last two decades, the volume of scholarly articles focusing on myocardial ischemia-reperfusion injury has grown exponentially, potentially causing an overload of information [10]. Many researchers find navigating through the extensive literature to discern research trends and topics challenging. Bibliometrics, the statistical analysis of written publications, serves to condense relevant data from the wealth of published research, offering insights into trends and identifying leading studies or scholars in the field. This methodology not only furnishes a clearer and more rapid comprehension of the subject matter for researchers but also highlights the plethora of research directions awaiting exploration [11].

Despite the abundance of studies on MIRI treatment, a comprehensive bibliometric analysis within this specific area is lacking to our knowledge. This investigation compiled literature from the Web of Science (WoS) core collection spanning from 2000 to 2023, employing CiteSpace and VOSviewer for visualization analyses to create a scientific knowledge map of the field. Our exhaustive literature collection and analysis aim to quantify and categorize publications within basic research, clinical research, preventive medicine, and other sub-disciplines, thereby sketching the current landscape and progression trends within MIRI treatment research. The outcomes of this study aspire to assist researchers in pinpointing leading journals, grasping popular topics, and identifying nascent research avenues to explore potential investigative segments. By delineating the present research scenario, focal points, frontiers, evolutionary trajectories, and developmental tendencies, we aim to furnish a comprehensive and systematic understanding for researchers delving into the arena of MIRI treatment trends.

## Materials and methods

Bibliometrics, which first appeared a century ago, was established as an independent discipline in 1969 and has since been widely applied to bibliographic research [12, 13]. The emergence of bibliometrics provides a reliable method for quantitatively studying the extant literature in a given field [14]. It enables the obtaining of valuable information such as authors, keywords, journals, countries, institutions, and references for analysis. Therefore, we analysed the present state of research in the field and assessed trends through the unique research method of bibliometric analysis [15]. Furthermore, with the aid of modern computer technology, we are capable of visualizing the results of our analyses, making them easier to comprehend [16]. Web of Science contains the highest quality journals worldwide and is recognized as the most influential database for scientific research, therefore this study used the WoS core database to search the literature [17–19].

We performed a data search on 1 October 2023 on the Web of Science Core Collection database. The subject term search formula was as follows, ((((TS = (myocardial NEAR/1 “reperfusion injur*”)) OR TS = (Cardiac NEAR/1 “reperfusion injur*”))) OR TS=(MIRI)) AND TS = (Therapeutic OR Therapy OR Therapies OR Treatment). Limitations: Publication date from 1 January 2000 to 1 October 2023. Both research articles and reviews, being valuable to the study, were included [20, 21]. A language restriction was applied, considering only English-language publications. The retrieved literature was then exported to text file format to facilitate further screening. Initially, duplicate search results were removed. Then, two authors independently reviewed all titles and abstracts, excluding those papers that were not relevant to the topic or whose article type was not an article or review, to confirm the selected literature. Disagreements, if any, were resolved by group discussion.

We chose VOSviewer and CiteSpace, the most popular research tools for bibliometrics, for our analysis [22, 23]. VOSviewer (version 1.6.19) was used to analyze the co-occurrence of information such as authors, countries, institutions and keywords, as well as network visualization mapping. CiteSpace (version 6.2. R4) was used to map the time zone view of published MIRI therapeutic area research articles and the biplot overlap of scientific and technical journals, as well as to generalize the year of keyword bursts.

## Results

### Overview of literature included

**Fig. 1:**
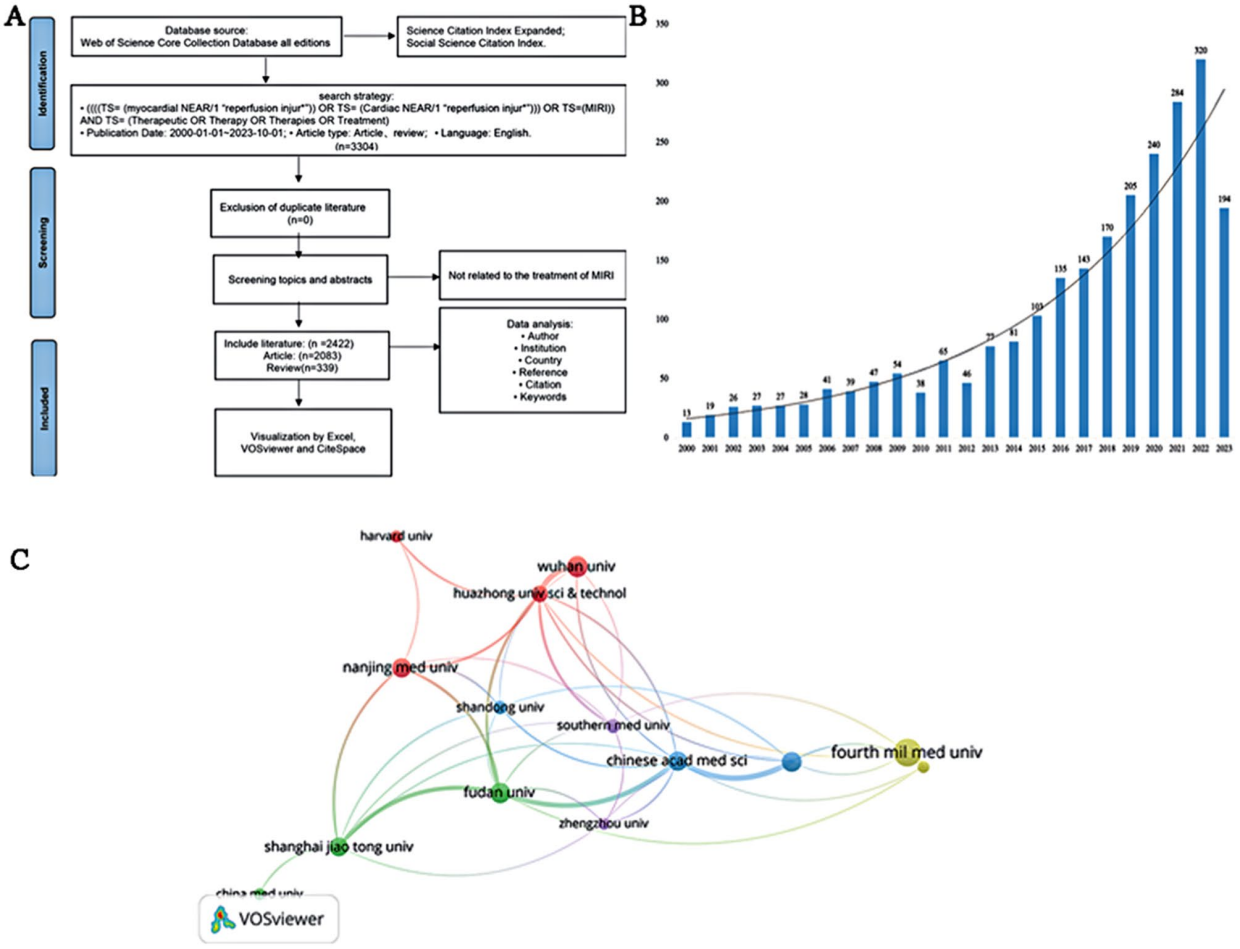
Literature search process, the changes in the annual publication volume and the inter-agency cooperation in this field. **A** Literature search and screening flowchart. **B** Trends in the quantity of articles published per year. **C** The map of scientific research cooperation relationship between the authored institutions of articles related to MIRI therapy. The thickness of the lines indicates the collaboration frequency

According to the search results, a total of 3,304 papers related to MIRI treatment were identified in the Web of Science Core Collection database from 1 January 2000 to 1 October 2023. The search and processing flow are depicted in Fig. 1A. The breakdown includes 2,083 treatises, 339 reviews, contributions from 13,228 authors, affiliations with 2,134 institutions, representation from 64 countries, and publications across 562 journals. Collectively, these works have accumulated up to 71,890 citations from 48,613 authors. We have illustrated the yearly publication volume in Fig. 1B, indicating a growing trend in research related to MIRI treatment. The number of publications for the year 2023 is expected to reach a new peak.

### Distribution of authors, institutions and countries

The literature included a total of 13,228 authors, with an average of 4 authors per article. A significant portion, 63.8%, of these authors contributed to just one article each. Table 1 showcases the five most prolific authors in the field; among them, Chattipakorn, Nipon stands out with the highest number of publications, totaling 33 articles. Additionally, Yang, Yang boasts the highest Average Citation per Publication, with a rate of 81.70.

**Table 1:** The five most published authors in the field

Rank	Author	Document	Citation	Average Citation/Publication
1	Chattipakorn, nipon	33	776	23.52
2	Chattipakorn, siriporn c,	31	730	23.55
3	Gao, Erhe	27	1544	57.19
4	Yang, Yang	23	1879	81.70
5	Yang, Jian	23	964	41.91

A total of 2,134 institutions were involved in research related to MIRI treatment after pointless nodes were excluded. Statistical and visual analyses targeted institutions with more than 34 publications, identifying the top 12 institutions. Additionally, the top ten institutions, in terms of publication numbers, are summarized in Table 2. The Fourth Military Medical University leads with 78 publications and 4,459 citations, demonstrating its significant influence in the MIRI treatment field. Notably, Harvard University, with only 34 papers, secures the second spot in citations (3,055) and has the highest average citation per publication. Figure 1C visualizes the collaboration among institutions, revealing close cooperation and frequent international exchanges within this field. The thickness of the lines indicates the collaboration frequency. For instance, Fudan University exhibits the closest cooperation with Shanghai Jiaotong University and the Chinese Academy of Medical Sciences, while Harvard University most frequently collaborates with Nanjing Medical University and Huazhong University of Science and Technology.

**Table 2:** The top five institutions in the field in terms of publications

Institution	documents	citations	Institution	documents	citations
Fourth Military Medical University	78	4459	Huazhong University of Science & Technology	47	1121
Wuhan University	59	1319	Southern Medical University	39	846
Fudan University	57	1307	Shandong University	38	641
Capital Med Univ	56	1203	Chiang mai University	36	779
Nanjing Med Univ	55	1352	Harvard University	34	3055
Chinese Academy Medical University	54	1293	China Medical University	33	633

The articles included in the study were distributed across 64 countries. We identified the top five countries based on the number of articles published, along with their respective numbers of articles, total citations received, and the average number of citations per publication, as detailed in Table 3.

**Table 3:** The top five countries in terms of the number of publications in the field

Rank	Country	Document	Citation	Average Citation/Publication
1	People’s Republic of China	1507	41,352	27.44
2	USA	458	26,457	57.77
3	Japan	100	5193	51.93
4	Germany	99	6365	64.29
5	United Kingdom	96	8010	83.44

**Fig. 2:**
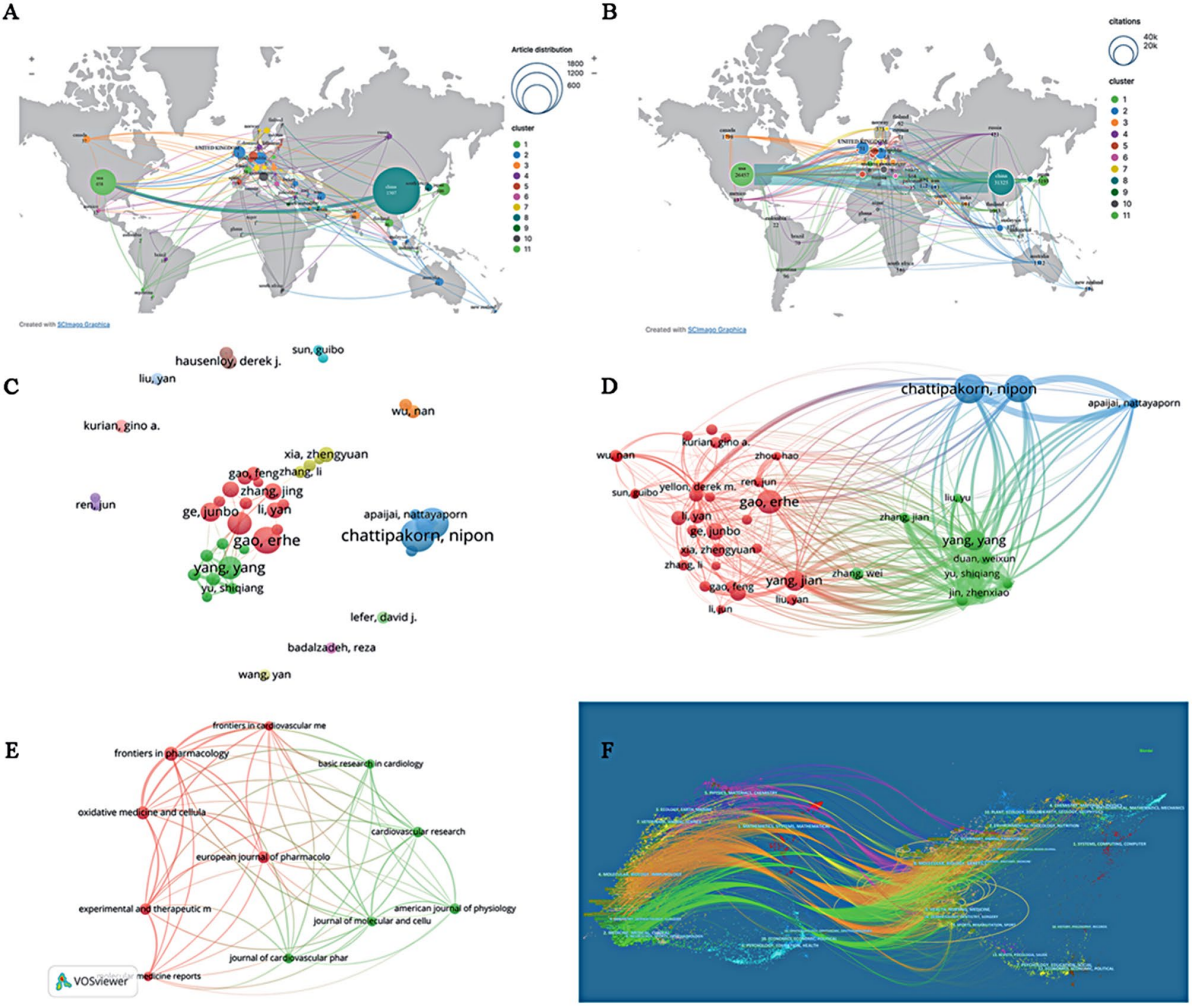
The relationship between the number of articles, citations and collaborations by country. **A-B** Global geographic distribution and web visualization maps of publications and citations by country/region. **C** Authors of articles greater than 10 and their collaborations. **D** Co-citations of authors of articles greater than 10. **E** Journals with more than 35 articles and their collaborations. **F** Dual-map overlay of journals related to the field of MIRI therapy

According to the statistics, China has the highest number of articles, reaching 1,507, surpassing the United States (485). Japan (100 articles), Germany (99 articles) and the United Kingdom (96 articles) followed. China has the highest citation frequency (41,352), followed by the US (26,457), and other countries have < 10,000 citations. However, the average citation/publication is highest in the UK (83.44), followed by Germany (64.29) and the US (57.77). China’s average citation/publication is the lowest among the top five countries in terms of the number of publications, only 27.44. In terms of the closeness of cooperation between countries, China and the United States have the most frequent exchanges with each other. And there is more communication between the United States and individual countries. The relationship between the number of articles, citations and collaboration by country can be seen in Fig. 2A-B. In summary, the articles are very unevenly distributed across countries, and the Matthew effect is very evident, with the majority of papers being produced by scholars from the leading countries.

### Distribution of authors and co-cited authors

In the past 20 years, a total of 13,228 authors have contributed to studies on MIRI treatment. For visualization and analysis purposes, we selected 42 authors who have published more than 10 articles. Chattipakorn, Nipon tops the list with 33 publications, followed by Chattipakorn, Siriporn C with 31 papers, and Gao, Erhe with 27 papers. The most cited author is Yang, Yang, with 1,879 citations, followed by Gao, Erhe with 1,544 citations, as indicated in Table 1. The highest average citations per publication were achieved by Yang, Yang, followed by Gao, Erhe, demonstrating the high quality of their articles and their relevance to the research areas of other scholars.

Figure 2C displays the results of VOSviewer’s visualization of author collaborations in this field’s literature. Connected clusters typically indicate collaborations and can provide future researchers with reliable indicators of potential partners. As illustrated in the figure, two closely-knit academic groups have emerged in the field. Additionally, the academic community represented by Yang, Yang, Gao, Erhe, and Ge, Junbo appears to be more frequently connected to the wider academic community. Meanwhile, researchers such as Badalzadeh, Reza and Wu, Nan are relatively isolated.

A co-citation relationship signifies the connection between authors that occurs when an article cites works by two or more authors. The nodes’ size in the graph correlates positively with the total citation numbers. The larger the node, the more citations the author has received, indicating greater influence. As depicted in Fig. 2D, the research interests of the authors are remarkably similar. Researchers from different clusters are indicated by nodes of varied colors. This study categorizes the authors into three main groups: Chattipakorn, Nipon, Chattipakorn, Siriporn C, etc. (blue); Yang, Yang, etc. (green); and Gao, Erhe, Yang, Jian, Ge, Junbo, etc. (red).

### Distribution of journals and most influential articles

The total number of papers from studies related to MIRI treatment included in this study has been published primarily in 562 academic journals. There are 11 journals with more than 35 publications each, with ‘*Frontiers in Pharmacology*’ having the highest number at 52, followed by ‘*Oxidative Medicine and Cellular Longevity*’ with 49 articles. These 11 journals account for 13.5% (446 out of 3,304) of the total number of publications (Table 4). The citation network diagram constructed from the above journals shows that ‘*Frontiers in Pharmacology*’ has substantial citation connections with journals such as ‘*Oxidative Medicine and Cellular Longevity*’ and ‘*Frontiers in Cardiovascular Medicine*’, as seen in Fig. 2E.

**Table 4:** Journals that have published more than 35 articles in the field

Rank	Journals	Document	Citation	Average Citation/Publication
1	Frontiers in Pharmacology	52	727	13.98
2	Oxidative Medicine and Cellular Longevity	49	1298	26.49
3	European Journal of Pharmacology	45	991	22.02
4	Experimental and Therapeutic Medicine	42	536	12.76
5	Cardiovascular Research	40	3291	82.28
6	American Journal of Physiology-heart and Circulatory Physiology	40	2948	73.7
7	Journal of Cardiovascular Pharmacology	37	702	18.97
8	Journal of Molecular and Cellular Cardiology	36	2070	57.5
9	Frontiers in Cardiovascular Medicine	35	267	7.63
10	Basic Research in Cardiology	35	1798	51.37
11	Molecular Medicine Reports	35	613	17.51

The relationship of this research topic to the primary research discipline is illustrated through a dual image overlay of the CiteSpace production journal. Figure 2F depicts each point representing a journal, with a map showcasing the size of journals on the left, and their citation count on the right [24]. The curves indicate validation lines, and the various colors represent different referencing relations. We identified two main citation paths (highlighted in green).

The top green path shows that articles from Medicine/Medical/Clinical journals typically cite works from Molecular/Biology/Genetics, whereas the bottom green path reveals that papers from Medical/Medical/Clinical journals mainly reference journals in the Health/Nursing/Medicine categories.

Furthermore, by sorting the search results, we highlighted the most influential papers, finding twenty-one papers that each have an average citation count exceeding 300. The most cited among these is ‘Myocardial ischemia-reperfusion injury: a neglected therapeutic target.’ We have summarized the fifteen most influential papers in Table 5.

**Table 5:** Most influential articles on MIRI therapy

Author(year)	Article	doi	citations
hausenloy (2013)	Myocardial ischemia-reperfusion injury: a neglected therapeutic target.	10.1172/jci62874	1505
Arslan(2013)	Mesenchymal stem cell-derived exosomes increase ATP levels, decrease oxidative stress and activate PI3K/Akt pathway to enhance myocardial viability and prevent adverse remodeling after myocardial ischemia/reperfusion injury.	10.1016/j.scr.2013.01.002	793
ibanez (2015)	Evolving Therapies for Myocardial Ischemia/Reperfusion Injury	10.1016/j.jacc.2015.02.032	688
kawaguchi (2011)	Inflammasome Activation of Cardiac Fibroblasts Is Essential for Myocardial Ischemia/Reperfusion Injury	10.1161/circulationaha.110.982777	616
ferdinandy (2007)	Interaction of cardiovascular risk factors with myocardial ischemia/reperfusion injury, preconditioning, and postconditioning	10.1124/pr.107.06002	583
matsui (2001)	Akt activation preserves cardiac function and prevents injury after transient cardiac ischemia in vivo	10.1161/01.cir.104.3.330	557
vinten-johansen (2004)	Involvement of neutrophils in the pathogenesis of lethal myocardial reperfusion injury	10.1016/j.cardiores.2003.10.011	514
Adlam(2005)	Targeting an antioxidant to mitochondria decreases cardiac ischemia-reperfusion injury.	10.1096/fj.05-3718com	512
turer (2010)	Pathogenesis of Myocardial Ischemia-Reperfusion Injury and Rationale for Therapy	10.1016/j.amjcard.2010.03.032	458
ren (2009)	MicroRNA-320 Is Involved in the Regulation of Cardiac Ischemia/Reperfusion Injury by Targeting Heat-Shock Protein 20	10.1161/circulationaha.108.814145	429
heusch (2020)	Myocardial ischaemia-reperfusion injury and cardioprotection in perspective	10.1038/s41569-020-0403-y	425
davidson (2019)	Multitarget Strategies to Reduce Myocardial Ischemia/Reperfusion Injury: JACC Review Topic of the Week.	10.1016/j.jacc.2018.09.086	408
hayashida (2008)	Inhalation of hydrogen gas reduces infarct size in the rat model of myocardial ischemia-reperfusion injury	10.1016/j.bbrc.2008.05.165	383
zhao (2019)	Mesenchymal stromal cell-derived exosomes attenuate myocardial ischaemia-reperfusion injury through miR-182-regulated macrophage polarization.	10.1093/cvr/cvz040	369
hausenloy (2005)	The reperfusion injury salvage kinase pathway: A common target for both ischemic preconditioning and postconditioning	10.1016/j.tcm.2005.03.001	361

### Keyword co-occurrence analysis

Keywords are the core vocabulary of a paper, which succinctly summarize the main content and are also frequently used throughout the article. We extracted 53 out of 2,610 keywords with a frequency of 50 or more for co-occurrence analysis in VOSviewer to explore trends and hotspots in the field of research. The top 20 keywords were also calculated and are presented in Table 6.

**Table 6:** The 20 most frequent keywords in studies related to MIRI treatment

Keyword	occurrences	total link strength	Keyword	occurrences	total link strength
cardiac ischemia-reperfusion injury	2066	7988	mitochondria	318	1487
apoptosis	755	3634	expression	309	1453
oxidative stress	541	2551	mechanisms	307	1488
heart	496	2394	inhibition	306	1515
activation	404	1986	protects	289	1462
cardiac protection	378	1896	pathway	287	1470
inflammation	358	1634	nitric-oxide	241	1056
infarction	343	1561	autophagy	194	930
cell death	342	1640	infarct size	187	812
animals	318	1451	disease	149	734

**Fig. 3:**
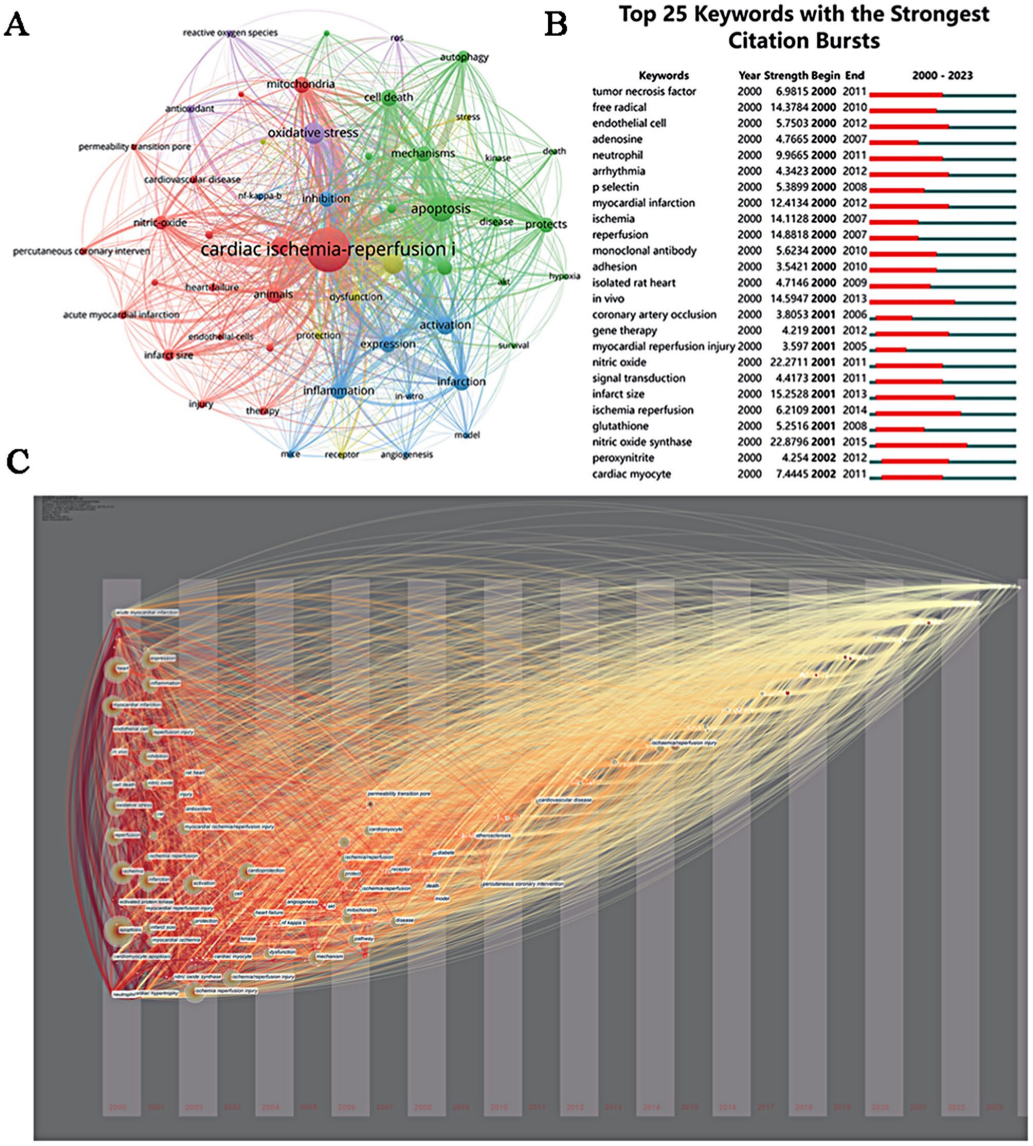
Keyword co-occurrence analysis and timezone map of keywords. **A** Co-occurrence analysis of 53 keywords cited by journals with frequency ≥ 50; **B** bursts of co-occurrence keyword map. The red line segment represents the time when the keyword emerges. The larger the strength value, the larger its burst strength section; **C** Timezone map of keywords. From the graph, we are able to see the chronological order of keyword appearances

In the visual representations, keywords are classified into four clusters as shown in Fig. 3A. Cluster 1 (colored red), the largest, primarily encompasses keywords associated with the MIRI mechanism and is centralized within the system, featuring terms like ‘cardiac ischemia-reperfusion injury’, ‘nitric-oxide’, ‘mitochondria’, and others in a dense distribution. Cluster 2 (green) concentrates on fundamental research into MIRI pathomechanisms, including keywords such as ‘apoptosis’, ‘mechanisms’, and ‘autophagy’. Cluster 3 (blue) pertains mainly to MIRI’s therapeutic mechanisms, highlighted by keywords like ‘activation’, ‘expression’, and ‘inflammation’. The fourth cluster (purple) is notably smaller, comprising a select few keywords like ‘oxidative stress’. Furthermore, an analysis of keyword citation bursts depicted in Fig. 3B revealed that ‘Tumor necrosis factor’ experienced the most substantial burst in popularity from 2000 to 2011. The significant citation bursts primarily occurred earlier on, suggesting that keyword citations in this field have reached a relative stability.

### Analysis of research hotspots

Keyword co-occurrence mapping reveals the dynamic evolution and research frontiers in specific search domains, achievable through keyword co-occurrence analysis [25]. The time-zone map of keywords examines the evolution of these keywords over time, unveiling changes and interactions within a particular research area [26]. As illustrated in Fig. 3C, the dynamic changes in the main keywords are displayed along a timeline.

Around 2002, “MIRI”, “acute myocardial infarction” and “oxidative stress” became research hotspots. Since 2005, “Mechanism” has become a hot research topic. Percutaneous Coronary Intervention” and “Atherosclerosis” have become hot research topics since 2009. Subsequent keywords appear less frequently and do not show up in the time zone graphs, probably because the main keywords did not change significantly over time. The above analysis of therapeutic research trends in MIRI may be divided into three main phases. The first phase was before 2002 and focused on the relationship between MIRI and inflammation. The second phase spans the period 2002–2009 and is characterized by a deepening of research into the therapeutic mechanisms of MIRI. In the third phase, from 2009 to the present, Percutaneous Coronary Intervention has grown rapidly and has become the treatment of choice and the most important research hotspot for coronary artery disease.

## Discussion

This paper uses bibliometric methods to assess the literature related to MIRI treatment from 2000 to 2023. From a modest 13 documents in 2000 to 320 documents in 2022, this study includes a total of 3304 representative papers. This significant increase is indicative of the growing scientific interest in MIRI treatment. The significant increase could be attributed to the recognition that early restoration of blood flow through reperfusion therapy is the gold standard for preserving the myocardium, although avoiding reperfusion injury remains a challenge [27–29].

The 3304 articles we included had a total of 13,228 authors, but most of them were occasional contributors. Core authors, comprising 274 (0.2% of all authors), produced no fewer than five articles each, contributing to 59.9% of the total literature. In contrast, 10,832 authors were involved in the publication of only 1 article in the literature, accounting for 63.8% of the total. It is demonstrated that the volume of postings is mainly concentrated in the head scholars, and the Matthew effect is very obvious. Authors with many publications often work together in teams. For example, the most renowned teams include Yang, Yang; Gao, Erhe; and Ge, Junbo. Their team has a large number of publications and citations, which contributes to a lot of research in the field.

The largest number of articles was issued by China, with 1,507 articles, or 45.6%. Next were the United States (13.9%), Germany (3.0%), Japan (3.0%) and the United Kingdom (2.9%). Eight of the top 10 institutions publishing articles on this topic are in China. In contrast, Chiang Mai University in Thailand and Harvard University in the United States made it into the top ten, but still ranked eighth and ninth, respectively. This again emphasizes the interest and importance Chinese scholars place on this topic. It is worth mentioning that Thai scholars (Chattipakorn, Nipon and Chattipakorn, Siriporn c.) ranked in the top two of the publication ranking with more than 30 publications, making a significant contribution to this field.

The results of the keyword timezone map show that the keywords in the literature related to MIRI treatment mainly exploded around 2002. Mechanisms of MIRI may be inflammation, oxidative stress, rapid pH correction, and intracellular Ca2 + overload [30, 31]. These are the reasons for the current clinical use of pharmacological treatments such as antioxidants, inflammation modulators and calcium channel blockers [32–35]. Understanding the pathophysiological mechanisms of MIRI can drive the advancement of novel clinical therapeutic options. In addition to mechanisms such as inflammation, oxidative stress, apoptosis, autophagy and calcium overload, in recent years, it has been suggested that mitochondrial damage and iron death also play a key role in the process of MIRI [36]. Since iron death is tightly related to mitochondrial metabolism, and mitochondrial energy metabolism during ischemia-reperfusion leads to excessive release of reactive oxygen species inducing iron death leading to myocardial injury.

Currently, anti-inflammatory, anti-oxidative stress, myocardial metabolism improvement and cardiomyocyte protection drugs are the mainstays of treatment for MIRI. However, the long-term use of these drugs has led to drug resistance or side effects in some patients. Therefore, non-pharmacological therapeutic options for MIRI are constantly being researched. After 2009, ‘nanocarriers,’ ‘heart regeneration,’ and ‘stem cells’ emerged as keywords across various literature but are not shown in the keyword rankings and time-zone maps because of the relatively small number of studies currently available.

With the rapid advancement of material science, nanocarriers have been constantly developed, and nano-drug carrier technology has become a hotspot in these years [37]. Nanocarriers are usually composed of organic and inorganic materials, which are immune to the body’s clearance of drugs and deliver them efficiently when they are loaded with drugs. Treatment of MIRI using nanotechnology focuses on mitigating myocardial injury by directly or indirectly reducing reactive oxygen species in the infarcted area [3]. Evidence from established laboratories confirms the far-reaching potential of nanocarrier technology in treatments against cardiac injury.

The conventional view in the scientific community is that the adult heart does not have the capacity for cardiomyocyte proliferation and that cardiomyocytes, like nerve cells, are considered permanent cells [38]. However, it has been found in recent years that the adult heart possesses some regenerative capacity in aging and pathological states [39, 40]. These neoplastic cells can be derived from pre-existing cardiomyocytes cardiac stem cells or precursor cells [41, 42]. Recent studies using small molecule drugs to promote cardiac regeneration have shown that cardiac regeneration can effectively compensate for the loss of cardiomyocytes after myocardial injury and thus reduce myocardial injury [43, 44]. These studies have expanded our understanding of the molecular basis of heart disease and continue to unearth key factors in cardiomyocyte proliferation. Heart regeneration and stem cells would be a great therapeutic strategy for MIRI patients in the future if they were used for clinical treatment. Thus, stem cells and heart regeneration have great potential in the treatment of MIRI and deserve further exploration.

Fortunately, the above novel treatment options for MIRI have progressed well in Phase II trials over the past decade or so, and Phase III trials are being rolled out to improve clinical care by applying laboratory results to humans [45]. With advances in clinical, AI, and industrial technologies, it is anticipated that MIRI treatments will soon offer increased patient benefits and reduced risks.

## Limitations

Although we rigorously completed the intended research process, inevitably this paper may have the following limitations. Firstly, we only retrieved English literature from the Web of Science core dataset database, which may affect the general applicability of the findings. Secondly, although we designed the search strategy and executed the process more tightly, we may have missed some literature as well. Thirdly, newly published literature may not have been cited enough times, leading to problems in keyword selection, which may not be accurate enough for future research directions. However, since the analysis relies on publications and their references, it inevitably faces several biases, such as publication and citation biases. Additionally, keyword-driven analyses can sometimes yield skewed results due to inaccurate keywords in some papers. Future research could delve deeper into these methodological issues or address broader topics.

## Conclusions

Using bibliometrics and visualization techniques, we have traced the progress, hotspots and frontiers of research in the field of MIRI treatment over the last 20 years. We also identified the scholars, institutions and countries that play an important role in the field. Keyword analysis showed the changing trends of research hotspots in the field and identified the main future research directions as protective strategies for MIRI such as nanocarriers, stem cells, and cardiac regeneration, and mechanistically autophagy and apoptosis are currently the latest hotspots. And we believe that with increased collaboration among researchers around the world, the variety of opportunities for interdisciplinary research collaboration will foster the development of high-quality research in this filed.

## Data Availability

No datasets were generated or analysed during the current study.
